# Asymmetrical Flow Field-Flow Fractionation Methods for Quantitative Determination and Size Characterization of Thiols and for Mercury Size Speciation Analysis in Organic Matter-Rich Natural Waters

**DOI:** 10.3389/fchem.2022.800696

**Published:** 2022-02-16

**Authors:** Isabelle A. M. Worms, Killian Kavanagh, Elodie Moulin, Nicole Regier, Vera I. Slaveykova

**Affiliations:** Environmental Biogeochemistry and Ecotoxicology, Department F.-A. Forel for Environmental and Aquatic Sciences, School of Earth and Environmental Sciences, Faculty of Science, University of Geneva, Genève, Switzerland

**Keywords:** AF4, DOM, reduced thiols, humic substances, colloids, mercury

## Abstract

Asymmetrical flow field-flow fractionation (AF4) efficiently separates various macromolecules and nano-components of natural waters according to their hydrodynamic sizes. The online coupling of AF4 with fluorescence (Fluo) and UV absorbance (UV) detectors (FluoD and UVD, respectively) and inductively coupled plasma–mass spectrometry (ICP-MS) provides multidimensional information. This makes it a powerful tool to characterize and quantify the size distributions of organic and inorganic nano-sized components and their interaction with trace metals. In this study, we developed a method combining thiol labeling by monobromo(trimethylammonio)bimane bromide (qBBr) with AF4–FluoD to determine the size distribution and the quantities of thiols in the macromolecular dissolved organic matter (DOM) present in highly colored DOM-rich water sampled from Shuya River and Lake Onego, Russia. We found that the qBBr-labeled components of DOM (qB-DOM) were of humic type, characterized by a low hydrodynamic size (*d*_h_ < 2 nm), and have concentrations <0.3 μM. After enrichment with mercury, the complexes formed between the nano-sized components and Hg were analyzed using AF4–ICP-MS. The elution profile of Hg followed the distribution of the UV-absorbing components of DOM, characterized by slightly higher sizes than qB-DOM. Only a small proportion of Hg was associated with the larger-sized components containing Fe and Mn, probably inorganic oxides that were identified in most of the samples from river to lake. The size distribution of the Hg–DOM complexes was enlarged when the concentration of added Hg increased (from 10 to 100 nM). This was explained by the presence of small iron oxides, overlapping the size distribution of Hg–DOM, on which Hg bound to a small proportion. In addition, to provide information on the dispersion of macromolecular thiols in colored DOM-rich natural water, our study also illustrated the potential of AF4–FluoD–UVD–ICP-MS to trace or quantify dynamic changes while Hg binds to the natural nano-colloidal components of surface water.

## 1 Introduction

Dissolved organic matter (DOM) is known to control the physicochemical speciation and, thus, the transport and bioavailability of Hg in aquatic environments (Ravichandran, 2004; Lavoie et al., 2019; Demarty et al., 2021). The binding of Hg to heterogeneous pedogenic DOM such as humic substances is expected to have a protective effect on aqueous organisms. However, Hg bioaccumulation has been shown to increase together with the concentrations of humic substances for copepods living in surface water of lakes with low dissolved organic carbon (DOC) (French et al., 2014). The binding of ionic Hg^II^ or its reduced form, Hg^0^, to humic substances has been shown to depend on the time of interaction (Wang et al., 2020). This kinetic limitation has significant consequences on the bioavailability of Hg to bacteria (Chiasson-Gould et al., 2014). Additionally, the presence or generation of inorganic particles may impact the transport and the bioavailability of Hg (Smith et al., 2002; Ravichandran, 2004). The formation of HgS, for example, is well recognized to depend on the sulfide/DOM ratio in anoxic soils or sediments and largely immobilized Hg (Liem-Nguyen et al., 2017; Liem-Nguyen et al., 2021), but the generation of nano-HgS was also pointed out to occur under oxic conditions (without sulfide) when DOM is present (Manceau et al., 2015a), and probably available for fish (Bourdineaud et al., 2019). Additionally, Hg can bind to various inorganic particle surfaces (Tiffreau et al., 1995; Bonnissel-Gissinger et al., 1999). Found as nano-colloidal species, particles are more likely to remain in surface waters and control the reactivity of trace metals bound to them despite their low concentrations (Lead and Wilkinson, 2006; Waeber et al., 2012; Cuss et al., 2020). Therefore, determination of the physicochemical speciation of Hg and the amount of Hg bound to DOM is of high relevance to understand its fate and bioavailability in surface waters.

As measurements of total dissolved Hg concentrations (<450 nm) were shown to be a poor indicator of Hg bioavailability, in particular when Hg is bound to humic substances or present in/associated with particulate forms (Ndu et al., 2018), different analytical techniques, such as diffusive gradient in thin films (DGT) and voltammetric sensor, were developed to measure labile Hg species *in situ* (Pelcova et al., 2014; Pham et al., 2015; Bratkic et al., 2019; Tercier-Waeber et al., 2021). For example, DGT labile Hg species were used as a proxy to assess the Hg accumulated by fish (Pelcova et al., 2017). Similarly, Hg measured by DGT was a valuable proxy for Hg bioavailability to bacteria under anoxic conditions, when Hg was bound to amorphous FeS, humic substances, or was present as nano-HgS (Ndu et al., 2018). A voltammetric sensor for *in situ* measurements of the dynamic Hg fraction was very recently used to measure low concentrations of Hg found in natural water (Tercier-Waeber et al., 2021). Unfortunately, the measurement of Hg species associated with the nano-sized components of DOM and small inorganic colloids was outside of the analytical windows.

The measurement of Hg bound to DOM can be performed using ion exchange, liquid–liquid extraction, or solid-phase extraction (Gasper et al., 2007). The latter was shown to be the most promising to determine Hg binding in aquatic systems under kinetic constrains (Miller et al., 2009). The use of stable isotopes for monitoring the exchange kinetics of Hg can also be used to estimate the kinetics of formation/dissociation of Hg complexes with DOM or at inorganic surfaces (Jiskra et al., 2014; Zhang et al., 2021). Recent developments using stepwise reduction of Hg combined with ligand exchanges and measured by cold vapor atomic fluorescence spectrometer (CVAFS) have provided information on the relative stability of Hg complexes and illustrated the kinetic limitation of Hg complexation by the strong binding sites of DOM (Liang et al., 2019). These techniques provided important information on the dynamics of Hg complexes under ambient conditions with implications on the bioavailability of bound Hg (Zhang et al., 2019; Zhao et al., 2019; Tang et al., 2020). However, they could not identify which specific component is involved in Hg binding, especially if nano-sized components of DOM and small inorganic colloids are present in complex mixtures.

Among the different functional groups present in DOM, thiols are considered as the strongest binding sites for metallic ions despite their low contents (Smith et al., 2002; Skyllberg et al., 2006; Catrouillet et al., 2015). Considering the importance of thiols and the sulfide phase in the speciation of soft metallic cations, such as Hg, their stability constants have been implemented in thermodynamic models (Liem-Nguyen et al., 2017; Liem-Nguyen et al., 2021; Smith et al., 2021). These studies highlighted the necessity to measure the thiol concentrations in the aqueous phase, at low concentrations. Currently available data on thiol contents in heterogeneous DOM were obtained by combining Hg LIII-edge extended X-ray absorption fine structure (EXAFS) and sulfur speciation obtained by X-ray absorption near edge structure (XANES) (Skyllberg et al., 2006; Manceau et al., 2015a; Manceau et al., 2015b; Song et al., 2018; Manceau and Nagy, 2019; Skyllberg et al., 2021). However, these techniques were not enough sensitive and required high amounts of DOM, making them inappropriate for use under commonly found conditions of surface waters. Nevertheless, these techniques revealed that the binding of Hg to the thiols present in heterogeneous DOM can be kinetically limited by the formation of other complexes involving carboxylate or amine groups (Jiang et al., 2015). Despite the improvements made in the measurement of the total thiol contents in surface water samples using fluorescent probes or mass spectrometry (Joe-Wong et al., 2012; Huynh et al., 2020; Liem-Nguyen et al., 2021), they do not discriminate the thiols present specifically in humic substances, for which the reactivity differed when compared to low-molecular-mass (LMM) thiols (Liang et al., 2019).

To date, the relationship between the thiols present in macromolecular DOM and the dynamics of Hg complexes formed with organic and inorganic nano-components has not been investigated in ambient water. This relays the difficulty of estimating the role of colloidal thiols involved in Hg binding (Richard et al., 2016b). Considering the analytical windows of the currently available techniques, the reactivity of inorganic small colloids can also be difficult to estimate because of the dynamic processes involved in their generation and their low occurrence in surface waters (Richard et al., 2016a; Hochella et al., 2019; Montano et al., 2019).

Asymmetrical flow field-flow fractionation (AF4) coupled online with fluorescence detection (FluoD), UV–visible detection (UVD), and inductively coupled plasma–mass spectrometry (ICP-MS) allows separating, according to their hydrodynamic size, and characterizing the composition of the nano-sized components. It can be used in complex environmental settings to follow changes in the size distribution of nano-colloids and the associated changes in the size speciation of trace metals (Stolpe et al., 2010; Stolpe et al., 2013; Neubauer et al., 2013b; Neubauer et al., 2013c; Cuss et al., 2018). For example, AF4 coupled online with FluoD, UVD and ICP-MS was successfully employed to quantify the size speciation of Hg, for instance, its binding to humic substances and the dynamics of the formation of Hg nanoparticles (1–100 nm) in pore waters, both involved in Hg mobilization from a contaminated soil subjected to flooding and redox oscillation (Gfeller et al., 2021). AF4 coupled online with UVD and ICP-MS was also used to follow the kinetics of Cd and Zn exchange by Ag in the 7-kDa peptide metallothionein (Liu et al., 2017). AF4 coupled with FluoD was also successfully employed to measure the molecular mass (MM) distribution of thiols produced by microalgae and naturally present in surface waters following fluorescent labeling (Mangal and Gueguen, 2015).

In this study, we thus explored the capability of AF4 coupled online with multi-detectors to: 1) measure the concentrations and size distribution of thiol groups in macromolecular DOM using monobromo(trimethylammonio)bimane bromide (qBBr) fluorescent labeling (referred to as qB-DOM) and 2) determine the Hg size speciation in the size range 1–450 nm for natural surface waters rich in humic substances and iron (Supplementary Figure S1). A methodology involving AF4–UVD–FluoD for the quantification of thiols was developed considering the potential interferences due to the fluorescence of humic substances on the determination of qB-DOM. We evaluated how the MM or size (hydrodynamic diameter, *d*_h_) distribution of qB-DOM can be influenced considering the retention of LMM fluorescent components during their injection due to membrane rejection and the potential inner-filtering effect of humic substances on fluorescence detection. Then, using AF4–UVD–ICP-MS, the size speciation of Hg was determined and related to the size distribution of other metals such as Fe, Mn, and Cu; the quantification of Hg–DOM species was also performed. Considering the very low Hg concentrations present in natural waters (Efremova et al., 2019), the samples were artificially enriched with Hg.

## 2 Materials and Methods

### 2.1 Chemicals and Reagents

Cysteine (Cys), glutathione (GSH), and qBBr were purchased from Sigma-Aldrich (St. Louis, MO, USA) as powders and used without further purification. All stock solutions (∼1 mM) were prepared from powders in deoxygenated Milli-Q water in a N_2_-purged tent and then preserved frozen at −20°C until use. Suwannee River natural organic matter (SRNOM, 1R101N), containing all the organic components isolated by reverse osmosis, was purchased from the International Humic Substances Society (IHSS, St. Paul (MN)) and stored at −20°C. A 500-mg L^−1^ stock solution of Suwannee River DOM (SRDOM) was prepared by dissolving the powder in Milli-Q water, slightly basified by the addition of saturated NaOH. Then, the solution was left to equilibrate in the dark at room temperature overnight and filtered on a 0.2-μm pore size filter (polyester sulfone; Millipore, Burlington, MA, USA). The SRDOM solution was preserved at 4°C in the dark.

The Hg analytical standard solution (1‐mgL^−1^ of mercury in 5% nitric acid; TraceCERT® CRM for AAS) was purchased from Sigma-Aldrich. Intermediate Hg solutions of 10 mg L^−1^ (49.9 μM) were prepared by dilution in 1% HNO_3_/0.5% HCl and used for ICP-MS calibration. The solution used for Hg enrichment of the DOM-rich water samples was prepared in 1% HNO_3_. Diluted acid solutions were prepared from Suprapur 67% HNO_3_, 35% HCl (Merck, Darmstadt, Germany), and Milli-Q water.

Ammonium nitrate (NH_4_NO_3_) and 2-[4-(2-Hydroxyethyl)piperazin-1-yl]ethane-1-sulfonic acid (HEPES), used as eluents for AF4, were obtained from Sigma-Aldrich, both prepared in Milli-Q water at 10 mM, pH 7.0. Before use, the solutions were filtered on 0.1-μm pore size polyvinylidene fluoride filters (Postnova Analytics, Landsberg am Lech, Germany). Sodium poly(styrene sulfonate) (PSS) standards of known MM, employed for the MM calibration of AF4 elution, were purchased from Postnova Analytics, dissolved in Milli-Q water at 1 g L^−1^, filtered with 0.1-μm pore size polyester sulfone filters (Millipore), and stored at 4°C. They were diluted in the eluent to 2–5 mg L^−1^ before use. Ultra-uniformed gold nanoparticles (UUGNPs) with a 10-nm core size (*d*_h_ = 19 nm) were used to determine the channel thickness in order to convert the retention time (*t*_r_) into hydrodynamic diameters. Stock suspension was purchased from NanoComposix (San Diego, CA, USA) and stored at 4°C. The suspension was diluted in proper eluent at a concentration of 0.5 mg L^−1^ after 30 s of homogenization using a vortex mixer.

### 2.2 Studied Area, Sample Description, and Preparation

#### 2.2.1 Natural Water Samples

Brown-colored DOM-rich natural waters were sampled in different locations of the transect River Shuya–Petrozavodsk Bay–Lake Onego (Karelia, Russia) during two field campaigns in March and June 2017. Detailed description of the sampling sites can be found in our previous study (Worms et al., 2019). The samples were filtered on site on a 0.45-μm regenerated cellulose filter before being transported and stored in the dark at 4°C for further analysis. The water samples are characterized by a high iron content and DOM was predominantly of fulvic type (Supplementary Table S1), as shown previously (Worms et al., 2019). The samples selected for analysis here corresponded to water taken from the Shuya River mouth and from the middle of the Petrozavodsk Bay at 3 different depths during the field campaigns in March and at 3 different locations but the same depth for June 2017. Lake Onego was sampled in June 2017. These samples are represented in gray in Supplementary Table S1.

#### 2.2.2 Labeling of Thiols by qBBr

The samples were spiked to reach 10 μM qBBr using small volumes of the qBBr stock solution and allowed to react for 2 h at 20°C. The reaction was stopped by decreasing the temperature of the samples to 4°C. To assess the membrane rejection of the adducts formed with low-molecular-weight thiols and of the unreacted qBBr on qB-DOM size distribution, qB-Cys and qB-GSH formed after the addition of an excess of cysteine or glutathione (20 μM) to qBBr solutions of different concentrations were used. SRDOM prepared in HEPES was also labeled and analyzed for its qB-DOM size distribution and quantity to validate the labeling procedure. Unlabeled samples were analyzed to determine the MM distribution of intrinsic fluorescent and absorbing components of DOM present in natural waters and to estimate how they can interfere with the qB-DOM signals.

#### 2.2.3 Mercury Addition and Total Mercury Measurements in the Samples

Mercury binding on the different nano-sized components was determined following Hg enrichment of natural samples using the 49.9-μM Hg stock solution prepared in 1% HNO_3_ and equilibration for 24 h in the dark at room temperature (20°C). The maximum concentration allowed for inorganic mercury by the European Environmental Quality Standard is 2.5 nM for drinking water (EU Directive 2013/39, line 226). This concentration is closed to conditions where Hg–DOM detection using AF4–ICP-MS could be limited (Gfeller et al., 2021). Thus, a slightly higher concentration of 10 nM and another one of 100 nM, more representative of highly contaminated waters, were adopted. A solution of SRDOM at 10 mg L^−1^ spiked with 100 nM Hg was also analyzed.

The total Hg remaining in solution after 1 and 24 h of equilibration (Hg_tot_) was determined with cold vapor atomic fluorescence spectrometer (CVAFS) coupled with a purge and trap system (MERX-T model III, Brooks Rand, Seattle, WA, USA). All samples were prepared following the Environmental Protection Agency (EPA) 1631 method in 40-ml glass vials with Teflon-lined septa caps. The stock calibration standard was purchased from Brooks Rand Instruments (4.9 μM Hg^II^ in 2% HNO_3_). The standards were prepared directly in glass vials and the samples diluted 25 times in Milli-Q water in glass vials. Both standards and samples were spiked with 100 μl of 2% BrCl and allowed to react overnight in the dark. Before the measurements, the excess of BrCl was reduced by the addition of 100 μl of hydroxylamine hydrochloride. The samples were then treated with 100 μl SnCl_2_ to convert all the Hg^II^ to elemental Hg^0^, capped, and directly analyzed using CVAFS. ORMS-5 water (National Research Council of Canada) was used as certified material. The obtained concentrations of Hg_tot_ are reported in Supplementary Table S1.

### 2.3 Determination of the Size Distribution of qB-DOM and Absorbing/Fluorescent Components by AF4–UVD–FluoD

Fractionation of the samples was carried out with AF2000 Focus (Postnova Analytics) coupled online to UVD and FluoD (Postnova Analytics). System control and data collection were performed using the AF2000 Control software (version 1.1.011, Postnova Analytics) and LC solution workstation software (Shimadzu, Kyoto, Japan). The trapezoidal channel was made of a 500-μm-thick spacer and a 300-Da cutoff polyethersulfone membrane (Postnova Analytics) as the accumulation wall. Samples were injected using a 1-ml sample loop at *V*_inj_ = 0.2 ml min^−1^ for 10 min. During this step, the sample was simultaneously injected, concentrated, and focused using *V*_foc_ = 3.4 ml min^−1^, *V*_xf_ = 2.7 ml min^−1^, and *V*_out_ = 0.7 ml min^−1^. After 1 min transition time, an elution step of 15 min at *V*_xf_ = 1.5 ml min^−1^ was followed by a 2-min linear gradient to *V*_xf_ = 0 ml min^−1^. The runs ended with a 10-min elution at *V*_xf_ = 0 ml min^−1^ to release the unfractionated material.

For the analysis of the qB-labeled samples, the fluorescence detector was tuned to *λ*_ex_ = 380 nm/470 nm, selective for qB-DOM adducts, and to *λ*_ex_ = 260 nm/470 nm, selective for the fluorescent components of humic substances (Fluo Humic). These wavelengths were adopted after the analysis of qB-Cys adducts and our samples with excitation–emission matrix (EEM) recorded on an LS 55 luminescence spectrometer (Perkin-Elmer, Waltham, MA, USA) using a 3-ml, 1-cm path length quartz cuvette. EEMs were generated by recording the emission spectra from 300 to 550 nm with 0.5-nm steps for excitation wavelengths that varied between 200 and 450 nm with 5-nm steps and corrected by subtraction of the EEMs obtained for Milli-Q water. For the size distribution of the absorbing components of humic substances (Abs Humic), *λ*_abs_ = 254 nm was selected.

The molecular masses of qB-DOM and humic substances were determined following the calibration of the AF4 elution with PSS standards. To this end, sodium PSS standards with MMs ranging from 1.1 to 10.6 kDa (Postnova Analytics) were injected and the log–log relationship between the retention time (*t*_r_) at peak maximum of the 254-nm fractograms and MMs was derived [log(MM) = 1.38 log(*t*_r_) + 0.21, *R* ^2^ = 0.995] and used to convert the *t*_r_ at peak maximum to MM for each sample. The AF4 elution theory was used to derive the relationship between *t*_r_ and hydrodynamic diameters (*d*_h_ = 1.065 *t*_r_) using UUGNPs to correct the channel thickness.

### 2.4 Characterization of the Association of Hg with Natural DOM Components by AF4–ICP-MS

To characterize Hg binding by the components of natural DOM and by the small inorganic colloids, AF4–FluoD–UVD was coupled online with ICP-MS (7700x model; Agilent Technologies, Santa Clara, CA, USA) as previously described using a He collision cell (ORS3) to reduce polyatomic interferences (Dublet et al., 2019; Worms et al., 2019; Gfeller et al., 2021). Fractograms were recorded in time-resolved analysis (TRA) mode using MassHunter software (Agilent Technologies) with a 0.5-s acquisition time for each isotope (^63^Cu, ^56^Fe, ^55^Mn, and ^202^Hg). The quantitative treatment for the Hg fractograms was performed by converting counts per second (cps) into parts per billion (ppb) using an external calibration of the ICP-MS instrument made of appropriate dilutions of the intermediate solution of 49.85 μM Hg, ranging from 0.5 to 10 nM, in the eluent containing 1% HNO_3_/0.5% HCl. A solution made in Milli-Q water and containing the same proportions of the different acids was used to rinse the tubing of the instrument between each measurement (Gfeller et al., 2021). The ICP-MS external calibrations were validated using digested phytoplankton certified reference material (IAEA-450, platinum in algae). Inter-batch calibrations were shown to be stable over several months, and our procedure showed less than 1% of deviation compared to the certified value. After the conversion of *t*_r_ into retention volume (*V*_r_, in liters), baseline correction and integration of the signals were performed using Origin®Pro 2019 (OriginLab, Northampton, MA, USA). Deconvolution of the Fe and Hg signals was performed using the same software in order to qualitatively relate the different forms of colloidal iron present in our samples and their interaction with Hg.

## 3 Results and Discussion

### 3.1 Evaluation of the Size Distribution and Quantification of the Thiol Groups in Natural DOM

**FIGURE 1:**
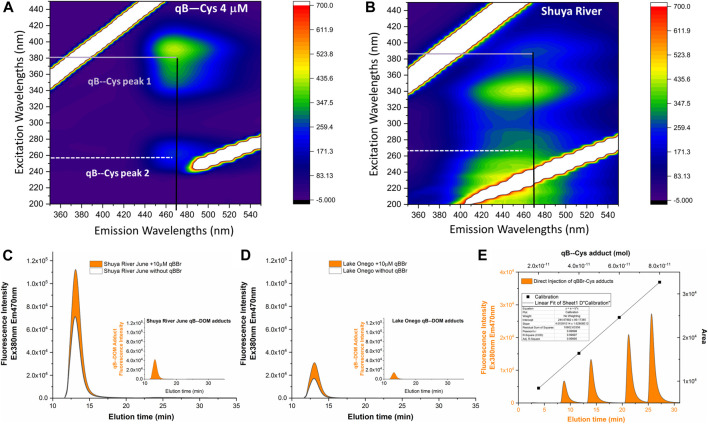
Illustration of the interference of the dissolved organic matter (DOM) present in natural water samples with measurements of the qBBr-labeled components of DOM (qB-DOM) by fluorescence. **(A**, **B)** Excitation–emission matrices of qB-Cys adducts **(A)** and DOM-rich water from Shuya River **(B)**. **(C**, **D)**Fractograms obtained by asymmetrical flow field-flow fractionation and fluorescence detection (AF4–FluoD) for Shuya River **(C)** and Lake Onego **(D)**. **(E)** Residual fluorescence of humic substances was subtracted from the fluorescence of labeled DOM to obtain the corrected qB-DOM size distribution [insets in **(C**, **D)**] further used to quantify the concentrations of qB-DOM using an external calibration performed using flow injection analysis (FIA) for increased quantities of qB-Cys in the eluent **(E)**.

Previous strategies using a titration of thiols by qBBr measured by batch fluorescence had limits of detection in the micromolar range for highly absorbing DOM, although the concentration of small (non-absorbing) thiols can be determined with a detection limit of 0.05 μM (50 nM) (Joe-Wong et al., 2012). The high detection limit for the determination of thiols present in DOM of pedogenic origin was due to the high fluorescence background of humic substances overlapping with the fluorescence of the qB adducts formed. Comparison of the EEMs of the qB-Cys adducts (Figure 1A) and the Shuya River water (Figure 1B) showed that the two *E*_x_/*E*_m_ wavelengths of qB-Cys overlapped with those of DOM, suggesting possible interferences of the fluorogenic materials present in our samples, indeed. The emission peak of qB-Cys adducts was shown to be less prone to interference of the humic fluorescent components than the one occurring in the UV region, where the fluorescence of humic substances was predominant. This second peak may refer to the one previously observed for the size distribution of qB-DOM, with *λ*_ex_ = 230 nm/470 nm, which was successfully used for the determination of thiols in algal growth media stripped of humic substances (Mangal and Gueguen, 2015).

Before qBBr labeling, the fractograms of the DOM-rich water samples exhibited a residual fluorescence at wavelengths corresponding to those used for the detection of qB adducts (Figures 1C, D, black lines). After the addition of qBBr, a significant increase in the intensity of the fluorescence signal was recorded, which can be attributed to the formation of qB-DOM adducts (Figures 1C, D, orange areas). Thus, the fractograms corresponding to the qB-DOM adducts (Figures 1C, D, insets) were obtained by subtracting the DOM fractograms obtained in the absence of qBBr from those recorded after the labeling of DOM with qBBr.

**FIGURE 2:**
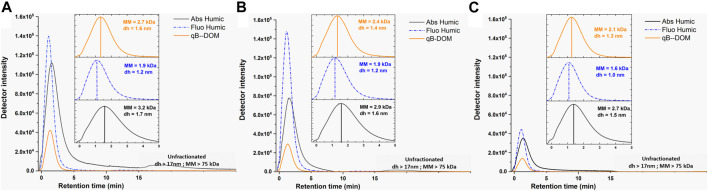
Fractograms obtained using asymmetrical flow field-flow fractionation (AF4) UVD–fluorescence detection (FluoD) for the UV-absorbing (*Abs Humic*; *λ* = 254 nm, *black lines*) and fluorescent humic-like (*Fluo Humic*; *λ*_ex_ = 270 nm/460 nm, *dotted blue lines*) components of DOM and for the monobromo(trimethylammonio)bimane bromide-labeled components of DOM (qB-DOM) adducts (*orange lines*) in waters from Shuya River **(A)**, Petrozadovosk Bay **(B)**, and Lake Onego **(C)**. *Insets* are zoomed in low retention times (*t*_r_ < 5 min) to facilitate the comparison of the MM and the hydrodynamic size, *d*_h_, obtained for the DOM components at peak maximum. Conversion of the retention time to molecular mass (MM) or *d*_h_ was performed by external calibration with poly(styrene sulfonate) (PSS) of known MM or AF4 theory corrected using UUGNPs of *d*_h_ = 19 nm, respectively. Release of unfractionated materials occurring when the cross-flow was swiched off corresponds to *t*_r_ > 16 min, corresponding to MM = 75 kDa and *d*_h_ = 17 nm, shown in *gray box*.

The qB-DOM adduct fractograms of the standard SRDOM and our samples were compared with those of the UV-absorbing (Abs Humic) or fluorescent (Fluo Humic) components (Figure 2). The MMs and size distributions of SRDOM (Supplementary Figure S2) were in the same range as those of the DOM-rich water samples (MM < 11 kDa, *d*_h_ < 4.3 nm; represented in the insets of Figure 2). Values at peak maximum were 2.5 kDa for the absorbing and 2.0 kDa for the fluorescent components of humic substances for SRDOM, thus a difference between absorbing and fluorescent components of Δ_MMabs-MMFluo_ = 0.5 kDa. The MM corresponding to the peak of distribution of the qB-SRDOM components (1.5 kDa) was lower than that corresponding to the fluorescent and absorbing components of DOM. For natural samples, similarly to SRDOM, higher values of MM were found for the absorbing (MM = 3.2–2.7 kDa) compared to the fluorescent (MM = 1.9–1.6 kDa) components: the Δ_MMabs-MMFluo_ were higher (from 1.3 to 1.1 kDa), suggesting a higher heterogeneity or agglomeration state of the humic components in our natural samples in comparison to SRDOM. Additionally, the MMs at the peak maximum of qB-DOM for natural samples were always intermediate between those corresponding to the fluorescent and absorbing components and varied from 2.7 to 2.1 kDa. The above results illustrated the heterogeneity in composition and size of the DOM components with different properties, from one environment to another. The evaluated hydrodynamic diameters at the peak maximum were always below 2 nm, as expected for humic substances, and it is encouraging that the qB-DOM measured here were part of this heterogeneous macromolecular pool of nano-sized DOM.

A comparison of the UV absorbance and fluorescence fractograms of DOM in all the studied samples revealed larger MMs and sizes for the absorbing components of DOM than fluorescent ones. Such a shift could be due to the impairment of fluorescence emission by an inner filtering effect, due to the shading of the excitation–emission lights by the absorbance of bigger-sized humic components (Senesi et al., 1991; Ohno, 2002). Similar considerations can apply to the fluorescence detection of qB-DOM. To verify whether the fluorescence intensities of 1.6 μM qB-Cys and 0.8 μM qB-GSH were altered by the presence of 10 mg L^−1^ SRDOM, we performed flow injection analysis (FIA) (Supplementary Figure S3). The results showed less than 5% error in the estimation of the qB adducts, implying that fluorescence detection was unaffected by the presence of the humic components in SRDOM.

Additionally, the shift toward apparent smaller-sized distribution parameters can be due to the contribution of the LMM components present as unreacted qBBr or as qB-DOM adducts and retained in the AF4 channel by membrane rejection. The retention of 8 μM qBBr (MM = 409.12 Da), qB-Cys (MM = 449.82 Da), or qB-GSH (MM = 635.54 Da) adducts was measured using two different eluents (Supplementary Figure S4). When HEPES was used as the eluent, low fluorescent peaks at retention times just after those corresponding to the void volume were found. These result showed that the small thiols labeled by qBBr and unreacted qBBr passed to a large degree through the AF4 channel membrane and were below the limit of size resolution under our conditions, but could contribute to an increase in the signal use for qB-DOM measurements. When NH_4_NO_3_ was used as the eluent, the fluorescence signals were identical to the eluent itself. Therefore, we concluded that, in this case, the fluorescence signal of qB-DOM was not influenced by the retention of LMM components. In addition, analysis of 13.3 mg L^−1^ SRDOM in the presence or absence of 4 μM of qB-GSH provided only a slight increase in the signal intensity (Supplementary Figure S4), showing that qB-GSH passed through the membrane even in the presence of macromolecular DOM. Thus, the retention of the LMM components could not account for the shift toward lower MM and size distribution of qB-DOM as compared with the MM and size distribution of the UV-absorbing components. Considering that the MMs of the qB-LMM used here were higher than that of the AF4 channel membrane cutoff (300 Da), the present results were consistent with those obtained by Zhou and Guo (2015), who reported an effective pore size cutoff of 1.9 kDa for this membrane.

Overall, the increase in fluorescence signal observed after the addition of qBBr in the samples was due to the reaction of qBBr with the thiol groups of the natural DOM components and can be used to estimate the thiols specifically contained in the pool of humic substances of macromolecules.

The qB-DOM fractograms were then integrated using OriginLab software and the area found were converted to qB-DOM quantities using external area-based calibration obtained by FIA of 100 μl qB-Cys standards (Figure 1). The calibration solutions were prepared by adding increasing concentrations of qBBr on a fixed cysteine concentration (20 μM) and diluted 10 times in the eluent to match the level of qB-DOM intensities of the samples. Under comparable conditions, a published study demonstrated that all the qBBr was converted to qB adducts when natural samples or standards presenting reduced thiols were in excess over the qBBr (Huynh et al., 2020). Maintaining an excess of thiols over qBBr decreased the possibility that unreacted qBBr may interfere with the measurement of the fluorescence signal.

Using qBBr labeling with the AF4–FluoD methodology, we found a thiol content of 4.8 ± 1.5 μmol g^−1^ (9.2 ± 2.7 μmol g^−1^ C) for SRDOM. Using titration with the ThioGlo-1 fluorescent probe, a value of 0.7 ± 0.1 μmol g^−1^ was calculated for SRDOM (Rao et al., 2014), although using a commercial kit, the content of thiols was estimated at 120 ± 9 μmol g^−1^ C (Smith et al., 2021) In both cases, the measurements were performed at a relatively high concentration of SRDOM (∼100 mg L^−1^). Thus, the large range in thiol content measured for this standard material can be attributed to the difficulties encountered in performing quantitative analysis of fluorescence measurements, when inner filtering and/or interferences of humic materials were not considered. Our methodology gave a thiol concentration closer but slightly lower than previously reported values using either X-ray absorption spectroscopy (XAS) or thiol titration by MS detection of residual qBBr, which both converged to 7.5 ± 0.4 μmol g^−1^ (Song et al., 2018; Huynh et al., 2020).

The loss of the LMM components of organic matter through (or on) the separation channel membrane was unavoidable for AF4 analysis, even for the lower cutoff membrane of 300 Da, as used in the present study (Zhou and Guo, 2015). The SRDOM recovery—estimated as a ratio between the peak area obtained after fractionation to the peak area found with FIA—was of 68% using Abs Humic as a surrogate of the DOM content. This contradicts the fact that most of the SRDOM [67% based on organic carbon (OC) measurements] was found to be of MM > 3 kDa using centrifugal filtration unit (CFU) (Song et al., 2018). However, in this specific study, the pH was lower than that in our study. Such lower pH could favor the aggregation of SRDOM components increasing in the same way as their retention by the filtration membrane. Similar experiments done with this CFU at neutral pH indeed showed lower DOC recovery (60%) (Bland et al., 2020). By considering the loss of the different components in the AF4 channel, a qB-DOM value of 7.0 μmol g^−1^ was derived for SRDOM using our methodology. This value, consistent with the literature (Huynh et al., 2020), indicated that the colloidal components of DOM contained similar thiol contents to the entire Suwanee River standard organic matter (OM) isolated by reverse osmosis.

The concentrations obtained for the qB-DOM adducts in natural water samples were below 0.5 μM, indicating the low thiol contents of natural DOM. Comparable concentrations of qB-DOM adducts in Shuya River samples, 0.25 ± 0.06 μM (March) and 0.35 ± 0.2 μM (June), were found. The concentrations of the qB-DOM adducts in samples from Petrozavodsk Bay were even lower: 0.09 ± 0.02 μM (March) and 0.14 ± 0.02 µM (June). In the open lake, the concentrations of the qB-DOM adducts fell further down to 0.05 µM. This decreasing trend in the concentration of qB-DOM adducts was consistent with the dilution process of DOM occurring in the river–lake transect. The thiol concentrations in natural DOM determined in the present study were lower than those reported in boreal aquatic environments, i.e., 1.16 µM in water from a stream and 0.96 µM in wetland dissolved fraction of pore water obtained by residual qBBr titration and mass spectrometry (Huynh et al., 2020). Such lower thiol concentrations were consistent with the rather limited biological activity found in Petrozavodsk Bay and Lake Onego during the sampling periods (Suarez et al., 2019). Much higher thiol concentrations have been reported in waters with higher microbial activity, leading to a larger production of LMM thiols (Mangal and Gueguen, 2015; Adediran et al., 2019; Mangal et al., 2020). For example, the thiol concentrations in high flowing water and marsh catchment of the Churchill River obtained by batch qBBr titration were much higher: 5.03 ± 1.94 and 21.0 ± 0.7 μM, respectively (Mangal and Gueguen, 2015). Furthermore, we did not expect freshly bio-produced reduced thiols to be preserved in our samples given their oxidation at neutral pH by organic matter (Chu et al., 2016). Finally, the low thiol concentrations found by AF4–FluoD could be due to the low amount of recovered material, as exemplified for SRDOM previously. Indeed, the recovery of the UV-absorbing components in our samples was of 36 ± 3% of the UV signal obtained by FIA. This latter can be overestimated due to the presence of iron oxides (Dublet et al., 2019), leading thus to an overestimation of material loss and cannot be directly used to correct the thiol-to-colloidal DOC ratios.

In natural samples, the thiol-to-DOC ratios were thus estimated using the humic contents determined by liquid chromatography–organic carbon detection (LC-OCD) (Worms et al., 2019) (Supplementary Table S1). The thiol content was 11.0 ± 1.4 μmol g^−1^ C independently of the sampling seasons or locations. Such low variability in the thiol/DOC ratio strongly suggest a similar source of a nano-sized (*d*_h_ = 1.4–1 nm) pool of thiols in the DOM. This suggestion is supported by the good correlation of qB-DOM with either the DOC or humic contents of the natural water samples (Supplementary Figure S5). The obtained thiol-to-DOC ratios were about twice lower than those found in boreal stream, 25.2 μmol g^−1^ C, but about 3.3 times higher than the one found in wetland pore water, 3 μmol g^−1^ C (Huynh et al., 2020). The obtained results implied that the nano-sized DOM could represent a “stable” pool of reduced thiols in DOM-rich surface water, and their role of Hg complexation should be considered.

**FIGURE 3:**
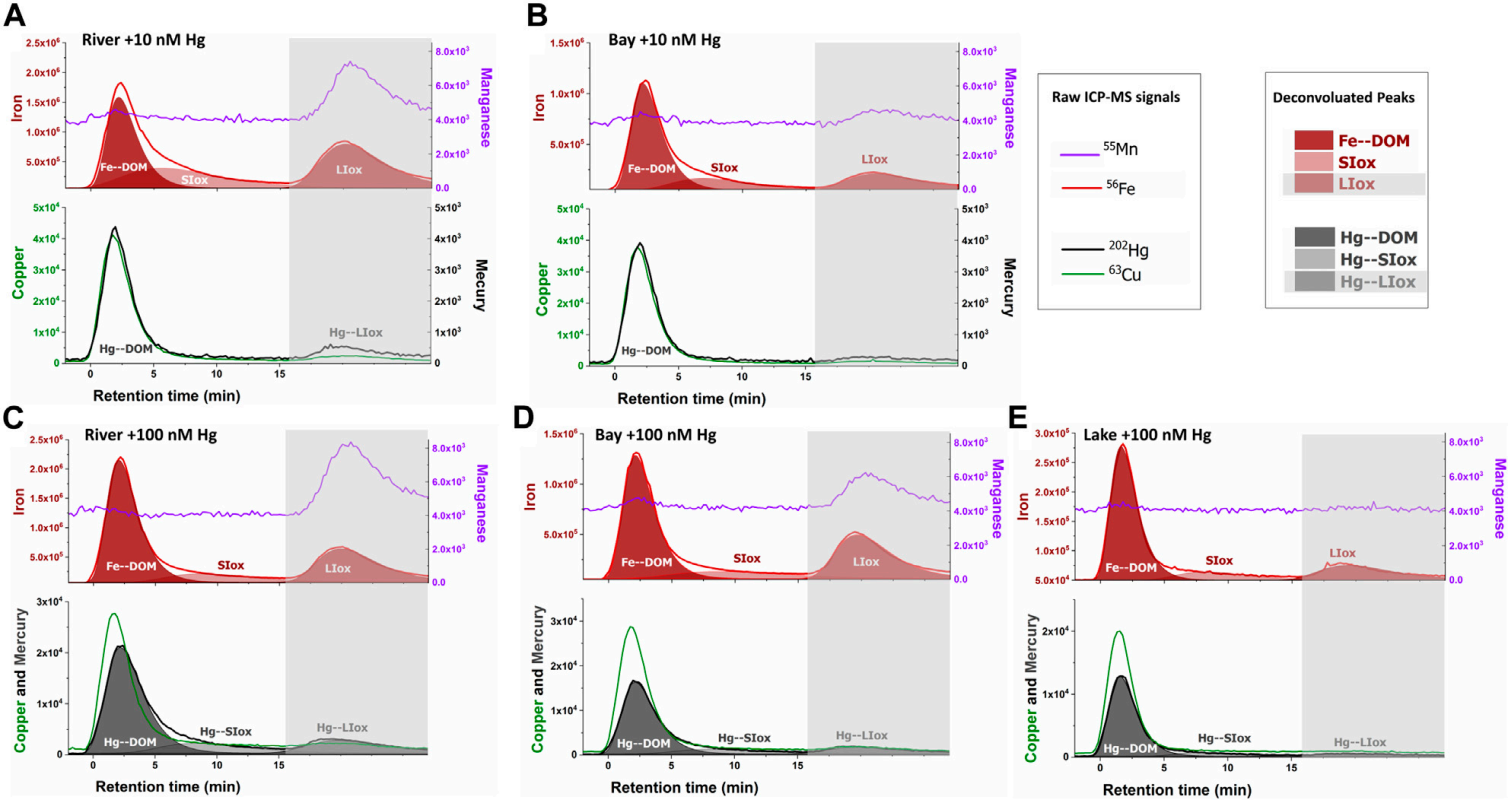
AF4–ICP-MS fractograms for iron (^56^Fe, *red lines*) and manganese (^55^Mn, *purple lines*) in *upper panels* and copper (^63^Cu, *green lines*) and mercury (^202^Hg, *black lines*) in lower panels for water samples taken from Shuya River **(A**, **C)** and Petrozadovosk Bay **(B**, **D)** enriched with 10 nM Hg **(A**, **B)** or 100 nM Hg **(C**, **D)** and Lake Onego enriched with 100 nM Hg **(E)**. The ^56^Fe signals were deconvoluted (*red areas*) using a 3-peaks “extreme” modeling in OriginPro software (Dublet et al., 2019), representing Fe bound to the nano-sized components of dissolved organic matter (DOM) (*Fe-DOM*), small iron oxides (*SIox*), and large iron oxides (*LIox*). LIox was eluted with the unfractionated material (*gray boxes*, >17 nm or 75 kDa). Deconvolution (*gray areas*) was performed on ^202^Hg peaks for samples enriched with 100 nM Hg.

Another aspect that should be considered is that the waters sampled were characterized by relatively high Fe/DOC ratios, 40.0 ± 4.3 µg Fe mg^−1^ C for Shuya River and 26.9 ± 4.3 µg Fe mg^−1^ C for Petrozavodsk Bay, whereas this ratio was 4.5 ± 0.2 µg Fe mg^−1^ C for Lake Onego (Supplementary Table S1). The Fe species recovered by AF4–ICP-MS (colloidal iron) corresponded to 60 ± 17% of the total dissolved iron measured by FIA, with lower values obtained in samples from Petrozavodsk Bay in June (35%). The nano-sized iron species in all the samples consisted of Fe–DOM complexes, small iron oxide nanoparticles (SIox), probably enrobed by humic substances and larger unfractionated iron colloids (LIox) using a deconvolution procedure (Figure 3, upper fractograms). The ^63^Cu peaks occurred with the absorbing DOM components and followed their size distribution (Worms et al., 2019). The recoveries for Cu were lower but less variable (42 ± 6%) than for iron, in agreement with the small size of the components complexing Cu, with optimized conditions for the elution of macromolecular DOM components. For the samples taken from Shuya River and Petrozavodsk Bay, Mn oxides co-eluted with LIox in the releasing peak (*d*_h_ > 17 nm) (Figure 3), but no co-elution of Mn was observed with DOM or SIox.

### 3.2 Distribution of Hg in the Nano-Sized Pool of DOM-Rich Natural Waters

To obtain insights into the MM and size distributions of Hg bound to the nano-sized components present in DOM-rich natural waters, the samples were enriched with 10 or 100 nM of Hg, equilibrated for 24 h, and then analyzed by AF4–UVD–FluoD–ICP-MS.

In the samples spiked with 10 nM Hg (Figures 3A,B), most of the eluted Hg (82 ± 9%) followed the elution profiles of Cu, a metal known to preferentially bound to humic substances. Hg also co-eluted with the UV-absorbing components with hydrodynamic size corresponding to *d*_h_ = 1.7 nm at peak maximum, but the peak shifted toward a higher size in comparison with the qB-DOM components (Supplementary Figure S6), showing the preferential association of Hg with DOM components of humic type (Hg–DOM). A low proportion of Hg (18 ± 9%) was associated with the unfractionated LIOx material. In the samples spiked with 100 nM Hg to mimic the conditions in a highly polluted environment, a similar proportion of Hg bound to LIOx (17 ± 4%) was measured. The percentages of Hg bound to small-sized components of DOM were comparable (83 ± 4%), but an increase of the retention of the Hg–DOM components was observed in samples with high DOC contents (Shuya River and Petrozavodsk Bay) (Figures 3C, D). In these samples, a higher hydrodynamic size at peak maximum (*d*_h_ = 2.2 nm) than that in Lake Onego (*d*_h_ = 1.7 nm) was found and larger size distributions of Hg components were observed (Supplementary Figure S6). These shifts in size distribution suggested that: 1) inter-macromolecular binding of the DOM components could occur in the presence of Hg (Worms et al., 2015) and/or 2) Hg could bind to small iron oxide (SIox) components co-eluting with Hg–DOM. The deconvolution of ^202^Hg signals revealed that Hg was distributed among the 3 main fractions: Hg–DOM and Hg bound to SIox and to LIox, indeed (Figures 3C–E). Based on our deconvolution procedure, the proportion of Hg–DOM accounted for 63 ± 7% of the fractionated Hg due to the binding of Hg to SIox.

It is worth noting that the addition of Hg resulted in changes in the size distribution of Fe species, especially SIox due to the slight acidification of the samples (Supplementary Figure S7). Additionally, the concentration of Hg measured by FIA indicated a loss of 61 ± 4% of Hg in the AF4 system for samples spiked with 100 nM Hg *versus* only 6 ± 14% when 10 nM was added. A decrease in the pH could favor the formation of large inorganic aggregates, which bind Hg, and could not be eluted from the AF4 channel and, thus, contribute to the loss of Hg (Neubauer et al., 2013a). However, since no significant change in the Fe recoveries in the samples was observed (±9%) (Supplementary Figure S7), we supposed that this could have only a minor contribution to Hg losses. To obtain further information on the Hg losses for samples spiked with 100 nM Hg, we performed measurements of the total dissolved Hg in water samples before injection in the AF4 channel (Hg_tot_) (Supplementary Table S1). The measurements revealed that only 32 ± 4% and 14 ± 9% of the initial Hg_tot_ concentrations were lost after 24 h of equilibration for the samples spiked with 100 and 10 nM Hg, respectively. These results demonstrated high Hg losses during the equilibration period, which were more pronounced at high Hg concentrations. A possible reason for such losses could be the reduction of Hg^II^ to volatile Hg^0^ by the DOM, which was shown to be kinetically controlled and dependent on the Hg/DOM ratio (Miller et al., 2009; Jiang et al., 2015). It could be suggested that part of the Hg^0^ produced by dark reduction over 24 h remained in our samples, measured by CVAFS, but did not elute from the AF4 channel.

Nevertheless, further optimizations would be needed, especially for an in-depth evaluation on how the largest inorganic colloidal species could account for Hg binding and how Hg^0^ could account for Hg speciation in artificially enriched natural samples.

**FIGURE 4:**
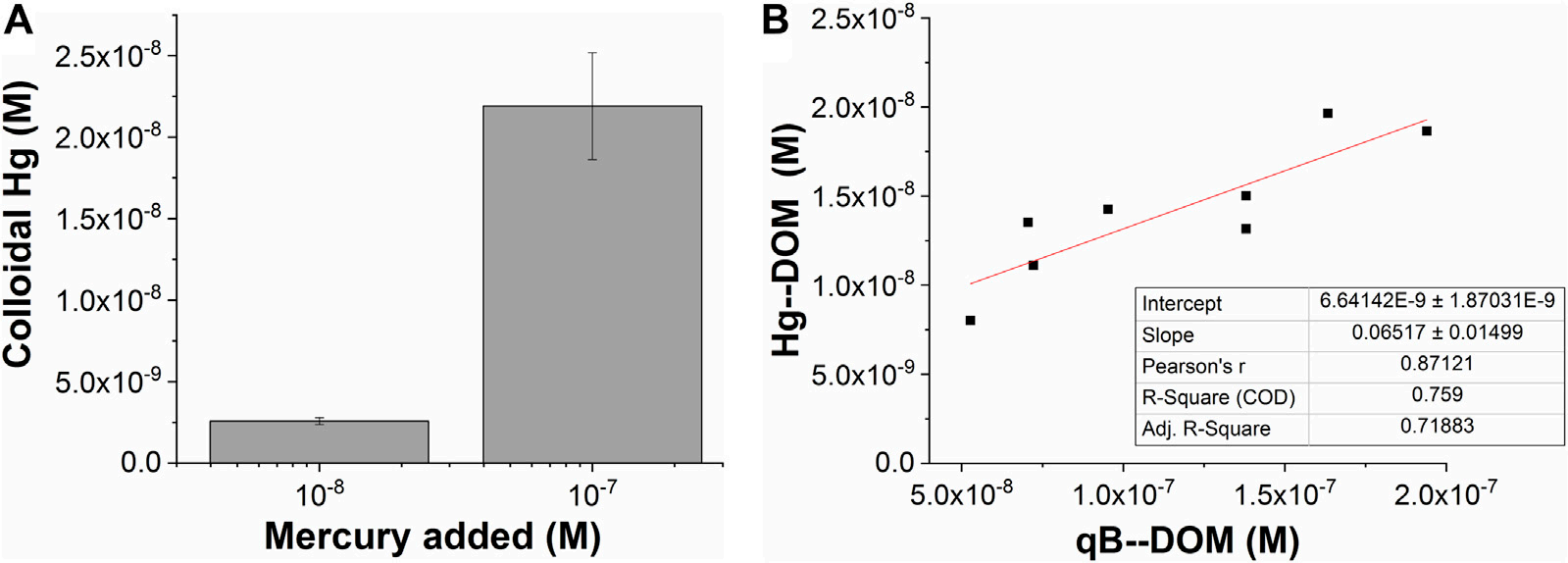
AF4–ICP-MS measurements of mercury recovered as a function of the total mercury added **(A)** and binding to dissolved organic matter (DOM) (Hg–DOM) related to qB-DOM adducts measured using AF4 with fluorescence detection (FluoD) when 100 nM Hg was added **(B)**.

When the losses during the equilibration of Hg with DOM after spiking were considered, the Hg recoveries after fractionation for samples spiked with 100 and 10 nM were in the same range, with 27 ± 7% and 30 ± 3%, respectively. The quantity of the measured Hg thus increased proportionally to the Hg added, with 10 times more Hg bound to the nano-sized components in samples from Petrozadovosk Bay and Shuya River at 100 nM than at 10 nM (Figure 4A). In addition, a good correlation between the concentrations of the Hg–DOM and qB-DOM measured for the samples enriched with 100 nM Hg was found (Figure 4B). Based on the available literature, we could expect that Hg–(S–DOM)_2_ complexes could be predominantly formed and that the reactive thiols of DOM can be saturated (Skyllberg et al., 2006). The Hg/DOM ratios (1.9–0.6 and 0.1–0.06 µg Hg mg^−1^ C at high and low Hg concentrations, respectively) used here were low enough to sustain the binding of Hg to high-affinity sites for isolated DOM (Haitzer et al., 2002; Miller et al., 2009). Consequently, the quantity of the Hg–DOM should correspond to about 50% of the values of qB-DOM, used as a measure of the reduced thiol contents in DOM; however, it only accounted for 27 ± 6% in our case.

Therefore, we suggest working under conditions where no saturation of macromolecular thiols by Hg is observed. The lower than expected proportion of Hg–DOM could be due to the following: 1) the presence of various dissolved components with higher affinity for Hg or with higher concentration, which were lost in the AF4 separation channel, for example considering the low pH of our samples and the formation of complexes between Hg and chlorides (the Cl^−^ concentration was 0.130 mM) (Supplementary Table S1). 2) The significant interaction of Hg–S–DOM species with the AF4 channel membrane preventing their elution. The binding of Hg to DOM components could change their charge or properties, which may favor the interaction with the membrane of the AF4 channel and result in a decrease of Hg recovery. A similar nonspecific interaction for AF4 fractionation was found at high Ag loading to the peptide metallothionein (Liu et al., 2017), leading to a decrease in the recovered metal. 3) The binding of Hg to inorganic colloid or Hg^0^ production and related losses, as discussed above.

Finally, the formation of Hg–(S–DOM)_2_ complexes should depend on the availability of proximal thiols within the macromolecular structure of humic substances or their capacity to promote inter-macromolecular binding. The measurement of the quantity of reduced thiols with the assumption that they formed Hg–(S–DOM)_2_ with high stability can be impaired by the formation of mixed Hg complexes involving carboxylates or amines, which were less stable but prone to be displaced during our analysis (Liang et al., 2019). Such type of mixed complexes was shown to be more sensitive to protonation, which entered in competition with Hg complexation (Haitzer et al., 2003).

### 3.3 Stability of Hg Bound to the Nano-Sized Components of Standard SRDOM

Since the DOM-rich natural waters contained a high proportion of colloidal Fe and their pH could be altered by Hg addition, we complemented our study by analyzing standard SRDOM samples prepared in HEPES buffer at pH 7.0. The samples were enriched with 100 nM Hg to give a ratio of 2 µg Hg mg^−1^ C sufficient to saturate thiols.

The measured Hg–DOM by AF4–ICP-MS (Supplementary Figure S8) corresponded to 44.2 ± 0.2% of the Hg added, corresponding to more than half of the qB-DOM content, measured to be 48 nM by AF4–FluoD in this case. To provide more complete information on the influence of the eluent type on the equilibrium of Hg binding with the DOM components during AF4 analysis, we compared the results obtained when HEPES instead of NH_4_NO_3_ was used as the eluent. High concentrations of NH_4_NO_3_ (10 mM) could be expected to favor the mobilization of Hg from low-affinity sites of SRDOM, as observed previously for carbonate-based ligands (Liang et al., 2019). The use of 10 mM HEPES as the mobile phase resulted in an increase in the proportion of Hg–DOM to 67.2 ± 1.7% of the added Hg. The partition coefficients (*K*_d_) of Hg obtained by the ultrafiltration of individual size fractions <30 kDa of the SRDOM were shown to be relatively comparable (Bland et al., 2020). Such lack of a clear size dependence of the Hg binding affinity could be explained by the presence of small-sized components (LMM of <3 kDa) with the same properties as the larger ones. This can rely on the isolation procedure of this standard. This supports our results showing that the values of thiol/C for macromolecular SRDOM components were identical to those of unfractionated DOM. Thus, considering that the recovery of SRDOM components (68%) was identical to the one of Hg (67.2 ± 1.7%) when HEPES was used as the eluent, it can be suggested that most of the Hg added was complexed by DOM, but that the use of NH_4_NO_3_ as the eluent displaced Hg from sites of low affinity during the analysis.

The results we obtained here for SRDOM are thus in line with the existing consensus that, after the saturation of thiols, other functional groups are involved in Hg complexation by DOM at neutral pH (Haitzer et al., 2003; Skyllberg et al., 2006). This provided evidence that the presence of other components or processes have affected the binding of Hg to the macromolecular thiols in our natural samples. This also suggested that the Hg–DOM we measured using AF4–ICP-MS for natural samples corresponded to the Hg bound to the relatively high-affinity sites of DOM. The fact that only a slight increase in the measured Hg–DOM was observed between 1 h when low-affinity sites must be involved (Jiang et al., 2015) and 24 h of equilibration in natural samples, but *t*_0_ (Supplementary Figure S9), also supported these hypotheses.

The influence of the type of eluent for Hg–DOM complexation could be used to design experiments that allow accessing the relative lability/stability of Hg–DOM complexes. The lability of metal–DOM complexes, together with their hydrodynamic size, is an important parameter for the evaluation of the bioavailability of toxic metals (Ndu et al., 2018; Bland et al., 2020); however, it is difficult to determine, in particular for heterogeneous samples. Given the heterogeneous nature of DOM with respect to size, the composition of its different components, and their reactivities, further in-depth studies regarding the relative lability of Hg–DOM complexes under fractionation conditions will be highly sought. No stand-alone technique is available to date for the assessment of the lability of Hg–DOM complexes. Therefore, the use of AF4–UVD–FluoD–ICP-MS, together with techniques providing the relative binding strength and the redox state of Hg (e.g., Liang et al., 2019), and determining the thiol components and their Hg binding capacity in the size range uncovered by AF4 (e.g., Kozyatnyk et al., 2016; Adediran et al., 2019) will be of high relevance. Additionally, the proportions of nano-HgS must be assessed as they can account for stable and small-sized mercury species certainly formed before 24 h, as recently discussed in Bourdineaud et al. (2019). This structural information could not be obtained by online elemental detection using ICP-MS. Combining size fraction collection with complementary techniques for material identification (iron or mercury nanoparticle composition) has been purposed (Dublet et al., 2019; Gfeller et al., 2021), but was limited by the low concentration of nanoparticles collected after AF4 fractionation.

## 4 Conclusion

In this study, we presented a methodology allowing the quantification of thiol groups in oxic water under the influence of a pedogenic DOM source. A combination of thiol labeling by fluorescent qBBr, separation of components by AF4, and online fluorescence detection was optimized for its uses to quantify the thiol contents in nano-sized macromolecular DOM together with their size distribution using Suwanee River DOM standard solutions. Neither the quantities of qB-DOM nor their size distribution was affected by the inner filtering properties of humic substances or by the presence of residual LMM fluorescent components. This methodology can be used to measure thiols under oxic/neutral conditions at relatively low concentrations (50 nM). The quantification and the characterization of the size distributions of Hg bound to macromolecules and nano-sized inorganic particles were obtained using AF4–ICP-MS. Using a signal deconvolution procedure, it was found that the spiked Hg was preferentially bound to macromolecular humic substances, with only a small proportion of Hg associated with inorganic nano-colloids. The measured Hg–DOM concentrations correlated well with the reduced thiol contents determined using AF4–FluoD. However, they were lower than those based on thiol contents. This result was explained by the potential reduction of Hg and the presence of other ligands in our samples impairing the binding of Hg to the thiols of macromolecules. We also showed that some Hg–DOM complexes can be displaced during their analysis by the NH_4_NO_3_ present in our eluent. These latter results open new opportunities to accessing the relative stability of the Hg–DOM complex when analyzed by AF4. The Hg–DOM contents we measured in DOM-rich natural waters using AF4–ICP-MS thus corresponded to Hg complexed to the high-affinity sites of nano-sized DOM.

## Data Availability

The raw data supporting the conclusion of this article will be made available by the authors, without undue reservation.
