# Supplementary material for: Asymmetrical Flow Field-Flow Fractionation Methods for Quantitative Determination and Size Characterization of Thiols and for Mercury Size Speciation Analysis in Organic Matter-Rich Natural Waters
Source: Front Chem. 2022 Feb 16;10:800696. doi: 10.3389/fchem.2022.800696 (PMC8888841; doi:10.3389/fchem.2022.800696)
Supplement: Supplementary file 1 [file DataSheet1.pdf]

Supplementary Material

Asymmetrical flow field-flow fractionation methods for quantitative determination and size characterization of thiols and for mercury-size speciation analysis in organic matter-rich natural waters

Isabelle A.M. Worms*, Killian Kavanagh, Elodie Moulin, Nicole Regier and Vera I. Slaveykova*

Environmental Biogeochemistry and Ecotoxicology, Department F.-A. Forel for Environmental and Aquatic Sciences, School of Earth and Environmental Sciences, Faculty of Science, University of Geneva, Uni Carl Vogt, 66, boulevard Carl-Vogt, CH-1211 Genève 4, Switzerland

***Correspondence:**
Corresponding Authors
isabelle.worms@unige.ch; vera.slaveykova@unige.ch

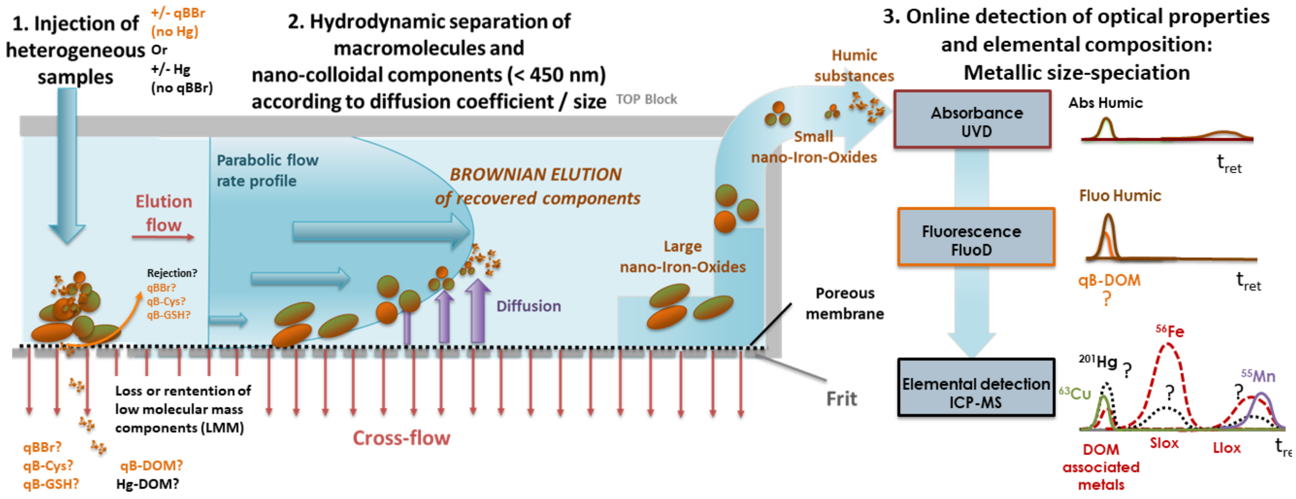

Figure S1. Shematic representation of the AF4 size-based separation of macromolecules and/or nanoparticles in heterogenous samples (< 450 nm) followed by their online detection using several detectors allowing their characterization and/or quantification. The UV-detector (UVD) was used to measure the size distribution of absorbing components mainly of organic origin, and attributed to the presence of humic substances. The fluorescence detector (FluoD) was used to measure intinsic fluorescence of colloidal organic material, also attibuted to humic substances, and the signals obtained after labeling of thiols using qBBr (+/- qBBr). For this study, the recovery and the membrane rejection of low molecular mass components were taking into account, as potential interferences for the measurements of macromolecular thiols. The nature and quantities of metallic species, either found bound to humic substances (macromolecular DOM, M–DOM) or as small or large inorganic nano-oxides (SIox, LIox) can be estimated using devonvolution of the ICP-MS signals. This procedure was used to estimate size-speciation of Hg, when the samples were enriched with this element (+/- Hg).

Table S1. Dissolved organic matter content (DOC, mg L^{-1}C), humic substances proportion (%, DOC) and total dissolved iron < 450 nm (Fe_{diss}, µg L^{-1}) of natural samples, their ionic composition (mg L^{-1}), and total mercury contents (Hg_{tot}, µg L^{-1}) following 1h and 24h of equilibration, after enrichment with 10 or 100 nM Hg. DOC, humic content and Fe_{diss} were obtained from (Worms et al., 2019). The ionic composition was measured by ionic chromatography following the method discribed in (Cossart et al., 2021). Total Hg concentrations in the samples (Hg_{tot}) were measured by CVAFS as described in the material and methods part.

		**DOM characterization by LC-OCD and iron content**									
		Shuya River	Bay S2	Bay S3	Bay S4		Shuya River	Bay S2	Bay S3	By S4	Lake Onego
	**d1**	**17.74**	**13.05**	**9.21**	**9.85**		**18.11**	**13.39**	**12.85**	**12.21**	**6.60**
**DOC < 0.45µm**	**d2**		**12.73**	**10.68**	**11.10**	/		**13.27**	**12.66**	**10.84**	**6.43**
**ppmC**	**d3**		**8.67**	**9.22**	**9.60**			**13.53**	**12.80**	**10.97**	**6.74**
	d1	0.79	0.45	0.25	0.25		0.65	0.27	0.37	0.29	0.03
**Fe < 0.45µm**	d2		0.46	0.31	0.33	/		0.30	0.39	0.29	0.03
**ppm**	d3		0.18	0.20	0.25			0.40	0.33	0.27	0.03
	d1	14.07	8.12	4.53	4.44		11.58	4.89	6.64	5.17	0.52
**Fe < 0.45µm**	d2		8.32	5.47	5.83	/		5.43	6.93	5.13	0.51
**µmol L^{-1}**	d3		3.19	3.61	4.45			7.23	5.85	4.81	0.58
	d1	**0.044**	**0.035**	**0.027**	**0.025**		**0.036**	**0.020**	**0.029**	**0.024**	**0.004**
**Fe/DOC**	d2		**0.037**	**0.029**	**0.029**	/		**0.023**	**0.031**	**0.026**	**0.004**
**mgFe mgC^{-1}**	d3		**0.021**	**0.022**	**0.026**			**0.030**	**0.026**	**0.024**	**0.005**
	d1	76.15	76.50	76.71	75.45		76.98	78.12	78.98	76.42	74.56
**% HS**	d2		78.63	75.93	76.41	/		77.62	79.07	77.37	75.33
	d3		74.11	73.38	75.98			79.64	76.88	77.53	75.45
		**Cations and anions composition (mg L^{-1})**									
	d1	**0.34**	**0.40**	**0.16**	**0.29**	/	**0.32**	**0.25**	**0.25**	**0.26**	**0.25**
K^{+}	d2		**0.19**	**0.27**	**0.31**			**0.21**	**0.27**	**0.23**	**0.25**
	d3		**0.39**	**0.29**	**0.28**			**0.27**	**0.37**	**0.26**	**0.20**
	d1	**20.59**	**22.87**	**11.24**	**18.91**	/	**20.59**	**16.76**	**17.70**	**17.44**	**16.53**
Na^{+}	d2		**14.14**	**16.73**	**18.83**			**15.06**	**17.07**	**16.59**	**17.20**
	d3		**23.24**	**18.27**	**16.86**			**17.57**	**16.71**	**16.79**	**13.95**
	d1	**3.31**	**3.32**	**3.31**	**3.31**	/	**3.31**	**3.31**	**3.31**	**3.31**	**3.31**
Mg^{+2}	d2		**3.31**	**3.31**	**3.31**			**3.31**	**3.31**	**3.31**	**3.31**
	d3		**3.31**	**3.31**	**3.31**			**3.31**	**3.31**	**3.31**	**3.31**
	d1	**3.50**	**4.86**	**4.26**	**4.90**	/	**3.50**	**3.65**	**4.00**	**4.54**	**5.22**
Ca^{+2}	d2		**3.71**	**3.73**	**5.12**			**3.75**	**4.05**	**4.49**	**5.22**
	d3		**5.17**	**4.53**	**4.64**			**3.91**	**4.02**	**4.43**	**4.95**
	d1	**1.30**	**1.44**	**0.31**	**1.11**	/	**1.09**	**0.97**	**1.05**	**0.94**	**0.86**
F^{-}	d2		**0.39**	**1.02**	**0.88**			**0.83**	**0.99**	**0.86**	**0.90**
	d3		**1.34**	**1.12**	**0.92**			**0.99**	**0.91**	**0.95**	**0.71**
	d1	**5.36**	**5.49**	**3.55**	**4.90**	/	**4.24**	**4.47**	**4.47**	**4.65**	**4.72**
Cl^{-}	d2		**4.28**	**4.39**	**5.97**			**3.97**	**4.59**	**4.67**	**4.90**
	d3		**5.85**	**4.75**	**4.61**			**4.55**	**4.62**	**4.46**	**3.89**
	d1	**2.3E-03**	**2.4E-03**	**2.7E-03**	**3.1E-03**	/	**2.7E-03**	**2.7E-03**	**3.2E-03**	**3.5E-03**	**4.0E-03**
Br^{-}	d2		**1.8E-03**	**2.1E-03**	**4.3E-03**			**3.1E-03**	**3.3E-03**	**3.3E-03**	**3.6E-03**
	d3		**3.2E-03**	**2.6E-03**	**2.3E-03**			**2.9E-03**	**2.8E-03**	**3.4E-03**	**3.2E-03**
	d1	**1.3E-02**	**1.2E-02**	**7.7E-03**	**8.1E-03**	/	**6.6E-03**	**7.2E-03**	**7.9E-03**	**n.a.**	**4.9E-03**
PO_4^{-3}	d2		**6.9E-03**	**9.7E-03**	**8.2E-02**			**8.5E-03**	**6.0E-03**	**6.1E-03**	**3.7E-03**
	d3		**7.8E-03**	**7.5E-03**	**6.3E-03**			**8.8E-03**	**n.a.**	**5.0E-03**	**5.3E-03**
	d1	**2.10**	**3.34**	**3.77**	**3.63**	/	**1.56**	**2.67**	**2.75**	**3.20**	**3.88**
SO_4^{-2}	d2		**2.99**	**3.02**	**4.33**			**2.63**	**2.76**	**3.19**	**3.98**
	d3		**3.93**	**3.43**	**3.42**			**2.71**	**2.86**	**3.24**	**3.94**
	d1	**0.94**	**1.07**	**1.28**	**1.17**	/	**0.52**	**1.12**	**0.99**	**1.08**	**1.25**
NO_3^{-}	d2		**0.98**	**1.66**	**2.97**			**1.09**	**1.07**	**1.16**	**1.20**
	d3		**1.24**	**1.26**	**1.38**			**0.96**	**0.85**	**1.17**	**1.30**
		**Total Hg after 1 h and 24 h of equilibration**							**(ppb)**		
	d1	**16.0**	**15.0**	**13.4**	**17.7**		**16.8**	**15.3**	**16.4**	**14.8**	**15.7**
100nM Hg t 1h	d2		**14.7**	**16.4**	**16.5**			**17.5**	**14.4**	**16.6**	**17.9**
	d3		**16.0**	**17.0**	**15.6**			**14.3**	**16.7**	**18.3**	**16.8**
	d1	**11.8**	**14.7**	**13.1**	**12.9**		**14.7**	**12.7**	**14.7**	**13.6**	**12.2**
100nM Hg t 24 h	d2		**14.7**	**14.8**	**15.5**			**13.8**	**12.9**	**14.5**	**12.2**
	d3		**12.9**	**14.8**	**13.9**			**15.5**	**13.0**	**14.5**	**13.2**
	d1	**1.9**	**1.7**	**1.7**	**1.7**		**1.7**	**1.8**	**1.7**	**1.8**	**1.9**
10nM Hg t 1 h	d2		**2.0**	**1.9**	**2.0**			**1.7**	**1.9**	**1.5**	**1.8**
	d3		**1.9**	**1.9**	**1.6**			**1.8**	**2.0**	**1.9**	**1.8**
	d1	**1.5**	**1.7**	**1.6**	**1.7**		**2.1**	**1.9**	**1.9**	**1.8**	**1.4**
10nM Hg t 24 h	d2		**1.6**	**1.7**	**1.5**			**2.0**	**1.6**	**1.9**	**1.7**
	d3		**1.4**	**1.8**	**1.7**			**1.8**	**1.8**	**1.7**	**1.4**

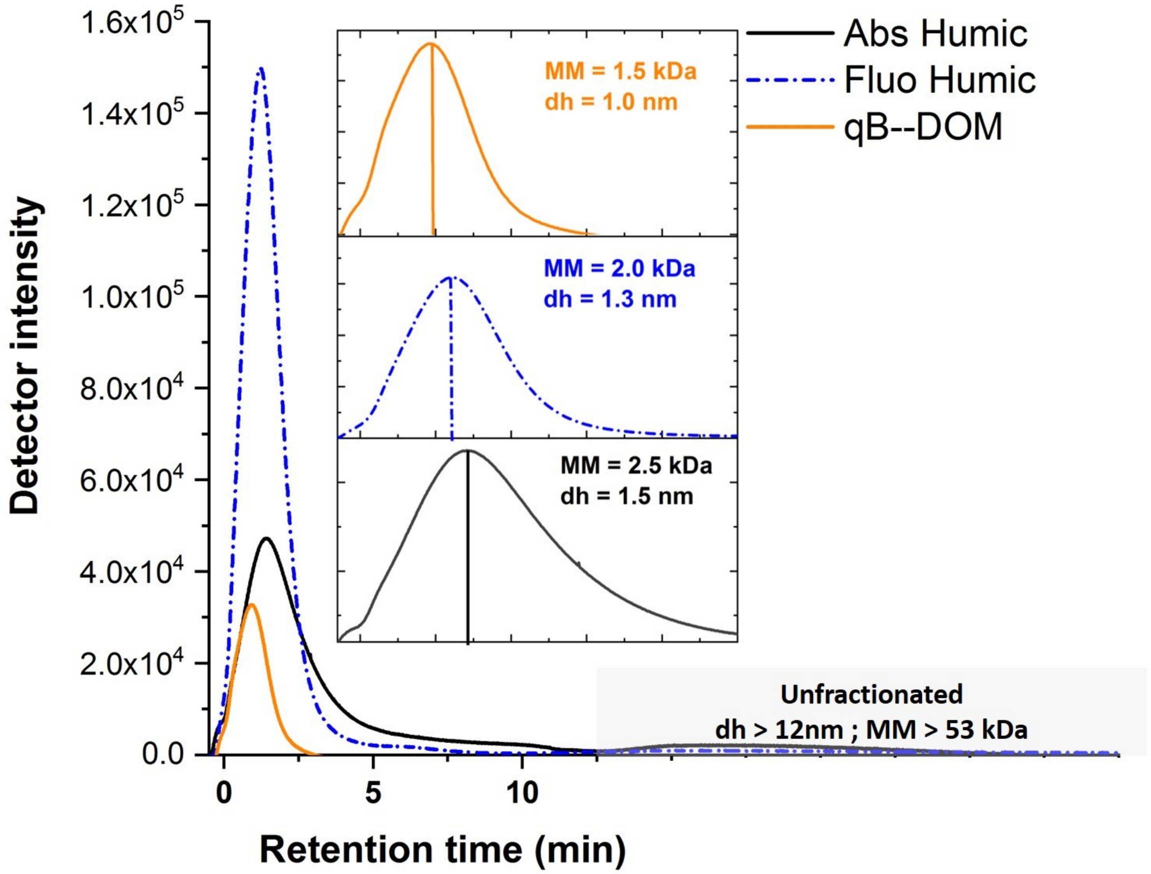

Figure S2. Comparison of fractograms obtained by AF4–UVD–FluoD for absorbing (Abs Humic; λ = 254 nm, black lines), fluorescent (Fluo Humic; λex = 270 nm/ λem = 460 nm; dotted blue lines) components of DOM and for qB–DOM adducts of SRDOM 10 mg L^{-1}. The inset illustrates normalized peaks for retention times t_r < 5 min, in order to facilitate the comparison of the MM and the d_h of DOM components at peak maximum; Retention times were converted into MM using external calibration with polystyrene-sulfonate standards or into hydrodynamic diameter using the AF4 theory with channel thickness corrected using UUGNPs (d_h =19 nm). Unfractionated material (gray box) elutes when the cross-flow is turned-off at t_r of 11.5 min, corresponding to MM of 53 kDa and d_h of 12 nm.

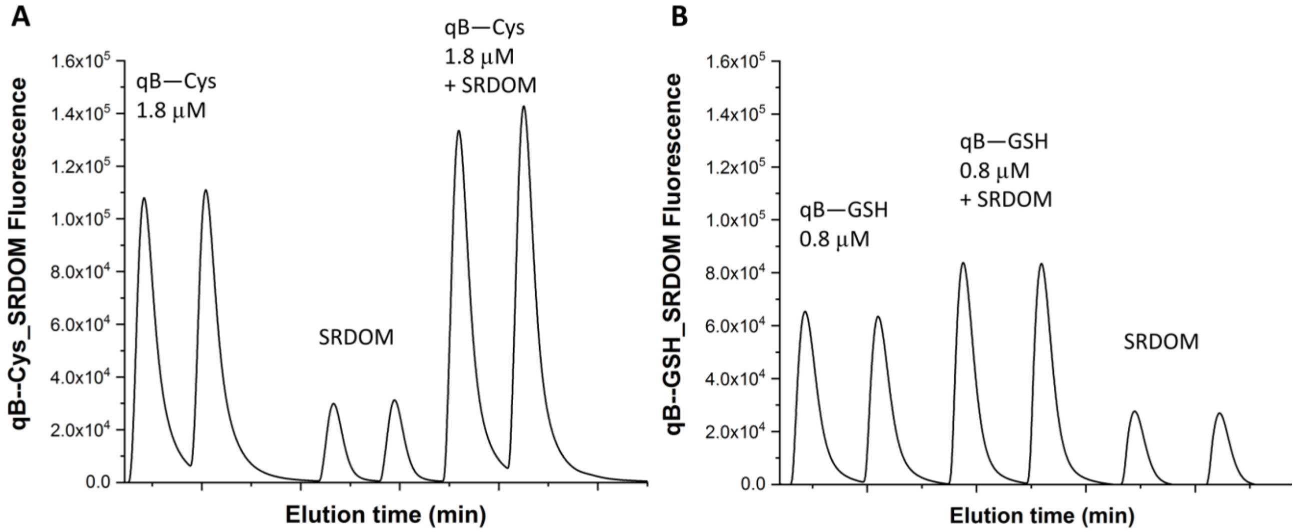

Figure S3. Evaluation of inner-filtering effect of colored SRDOM (10 mg L^{-1}) on qB–DOM determination using Cysteine (qB–Cys, A) or Glutathione (qB–GSH, B) solutions containing 1.8 μM and 0.8 μM of qBBr respectively, obtained by flow injection analysis (no cross-flow)

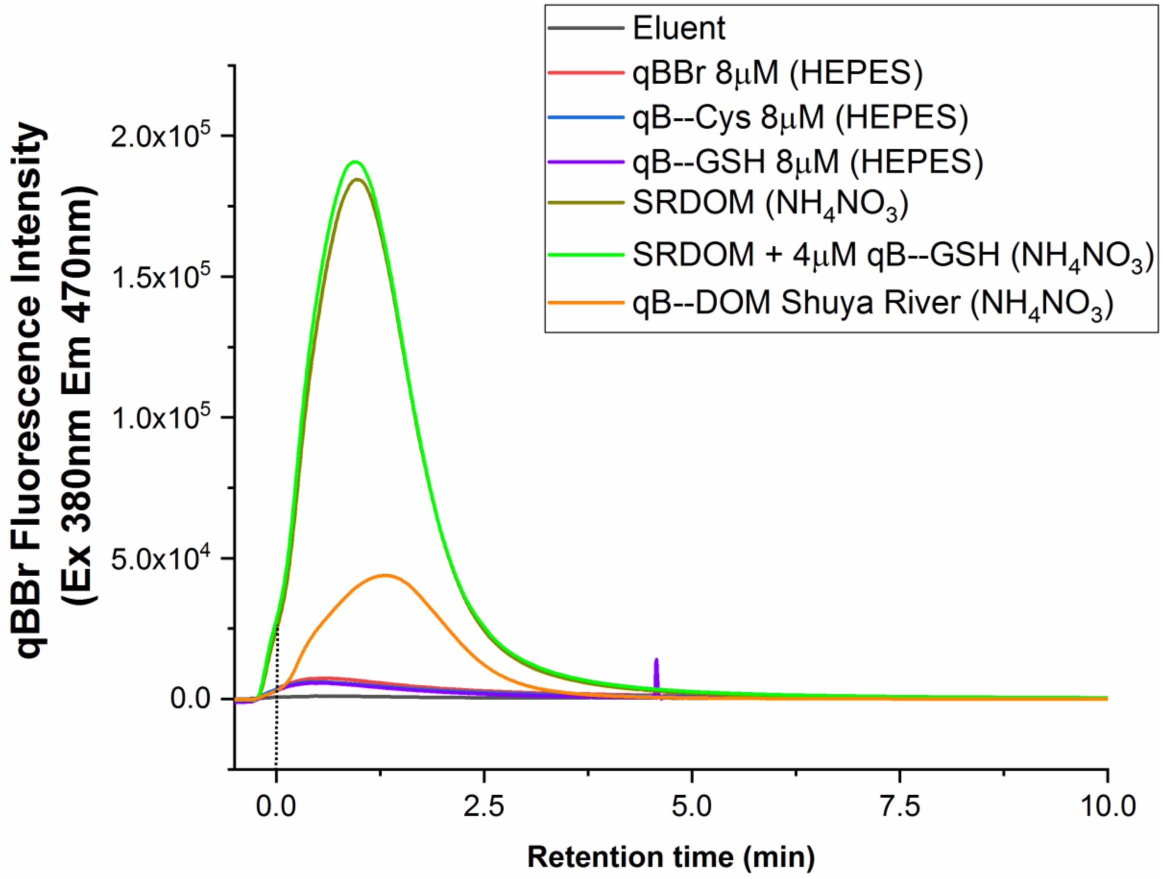

Figure S4. Influence of the eluent type on the retention of qBBr and qB–LMM adducts in the AF4 channel. Eluents: HEPES or NH_4NO_3 10 mM pH 7.0. Separation conditions are the same as for qB–DOM adducts measurement. The qBBr, qB–Cys and qB–GSH profiles were not measurable (not shown) when NH_4NO_3 was used as eluent, indicating that they were passing through the membrane and may not influenced the qB–DOM measurements. 10 mM NH_4NO_3 was thus selected for the measurement of qB–DOM and AF4–ICP-MS analysis. The presence 4 µM qB–GSH does not affect the fluorescence fractograms of 13.3 mg L^{-1} SRDOM. The fractogram of qB–DOM of Shuya River was also plotted for comparison. The dotted line indicates the retention time t_r0, at “void volume”.

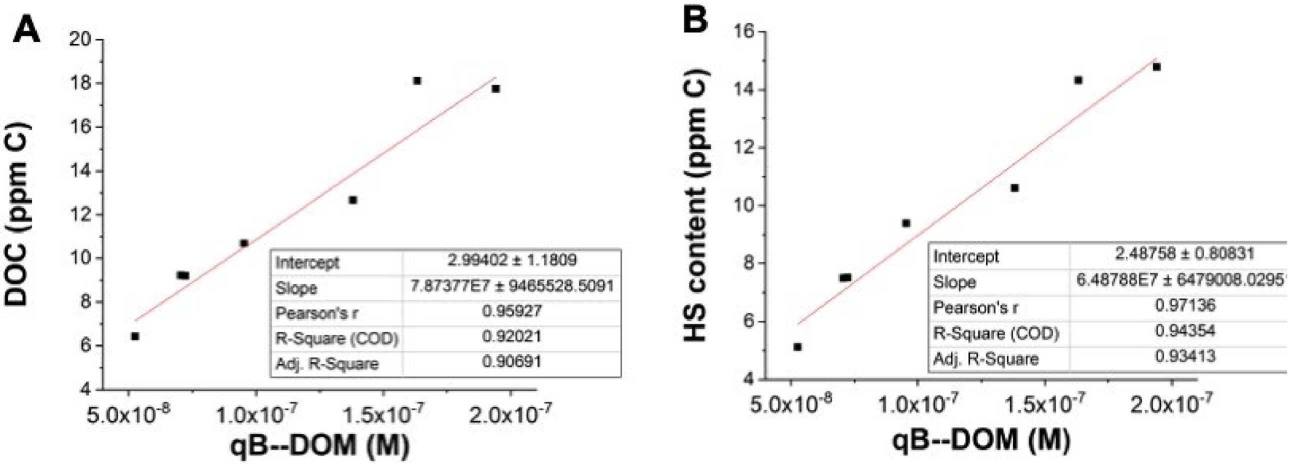

Figure S5. Correlations between total dissolved organic carbon (DOC, A) or humic content (HS, B) with qB–DOM measured in several samples from river to lake transect. DOC was measured after 0.45-µm filtration of the samples. The content of humic substances was determined using LC–OCD. (Worms et al, 2019)

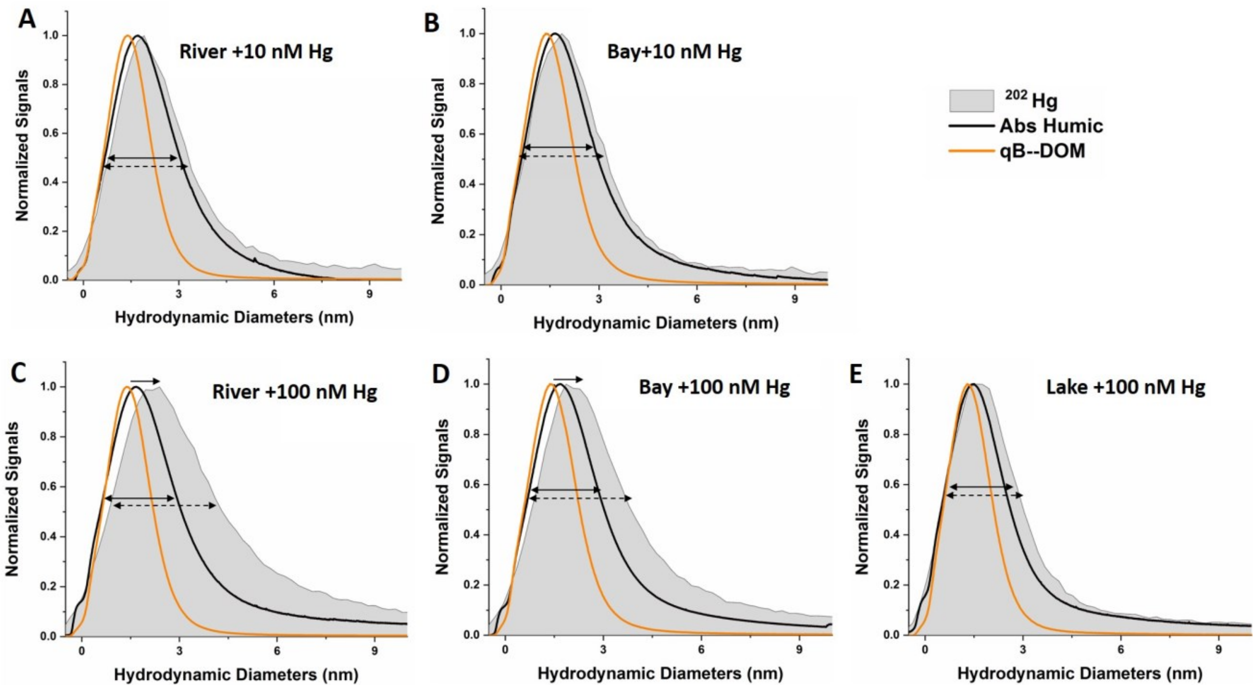

Figure S6. Comparison of hydrodynamic size distributions obtained by AF4–UVD–FluoD–ICP-MS of different components: UV-absorbing (Abs Humic, black lines), qB–DOM adducts (qB–DOM, orange lines) and Hg–DOM (^{202}Hg, grey areas), of water sampled from Shuya River (A, C), Petrozadovosk Bay (B, D), and Lake Onego enriched with 100 nM Hg (E). Samples were enriched with 10 nM Hg (A, B) or 100 nM Hg (C-E). The intensity of the signals was normalized to the one obtained at peak maximum. Small differences in size distribution between UV-absorbing components and mercury traces were observed at low concentration of Hg (10 nM) as indicated by the two double-arrows, but a shift in the size at peak and an enlargement of size distribution of Hg species were observed at high concentration of Hg (100 nM), when they have a high content in DOC.

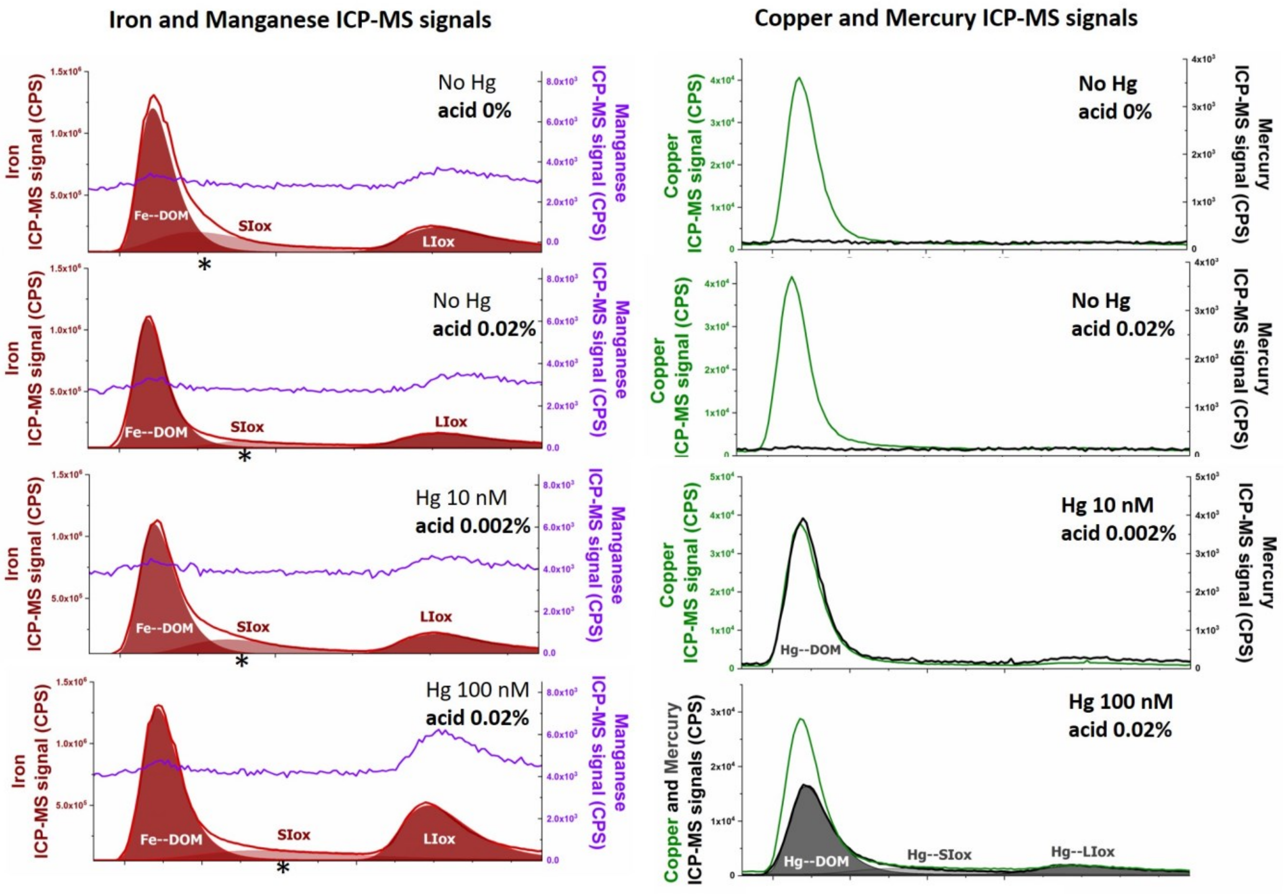

Figure S7. Effect of acidification (0.02%), due to the presence of HNO_3 in the spiking solution of Hg (49.85 μM), compared to spiked waters (10 and 100 nM Hg) on colloidal metal (^{55}Mn, ^{56}Fe, ^{63}Cu, ^{202}Hg) fractograms measured by AF4–ICP-MS for a water sample taken from Petrozadovosk Bay. The distribution of ^{56}Fe obtained by signal deconvolution shows a predominant effect of acidification on *SIox* hydrodynamic size (*). Addition of 10 nM Hg, together with 0.002% leads to a small shift. The presence of 100 nM Hg increased the recovery of *LIox* and colloidal Mn as compared with slightly acidified samples. The iron recovery obtained by comparison with flow injection analysis (36% for these analysis), only varied by 9% despite changes in acidity. The iron recovery of replicates analysis of this Bay sample was of 33 ± 11% and of 38 ± 9 % when 10 or 100 nM Hg, respectively.

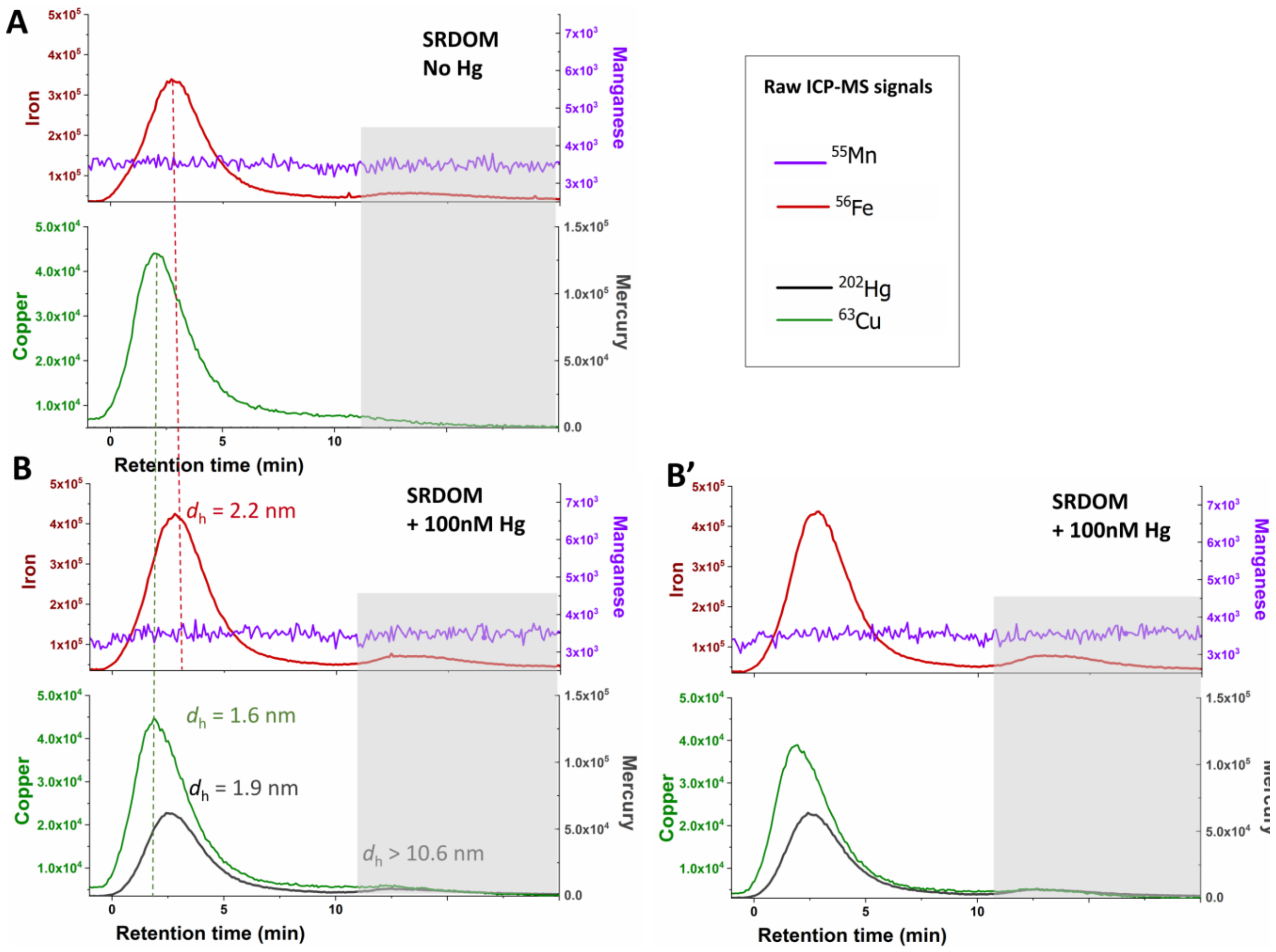

Figure S8. Comparison fractograms obtained by AF4–ICP-MS analysis of colloidal metal (^{55}Mn, ^{56}Fe, ^{63}Cu, ^{202}Hg) for 10 mg L^{-1} SRDOM without (A) or after addition of 100 nM Hg (B, B’). The hydrodynamic diameters d_h, at peak maximum of metal bound to DOM are indicated. Release of unfractionated materials (gray boxes) occurs when the cross-flow is turned-off, started to elute at t_r = 10 min corresponding to MM = 38 kDa and d_h = 10.6 nm.

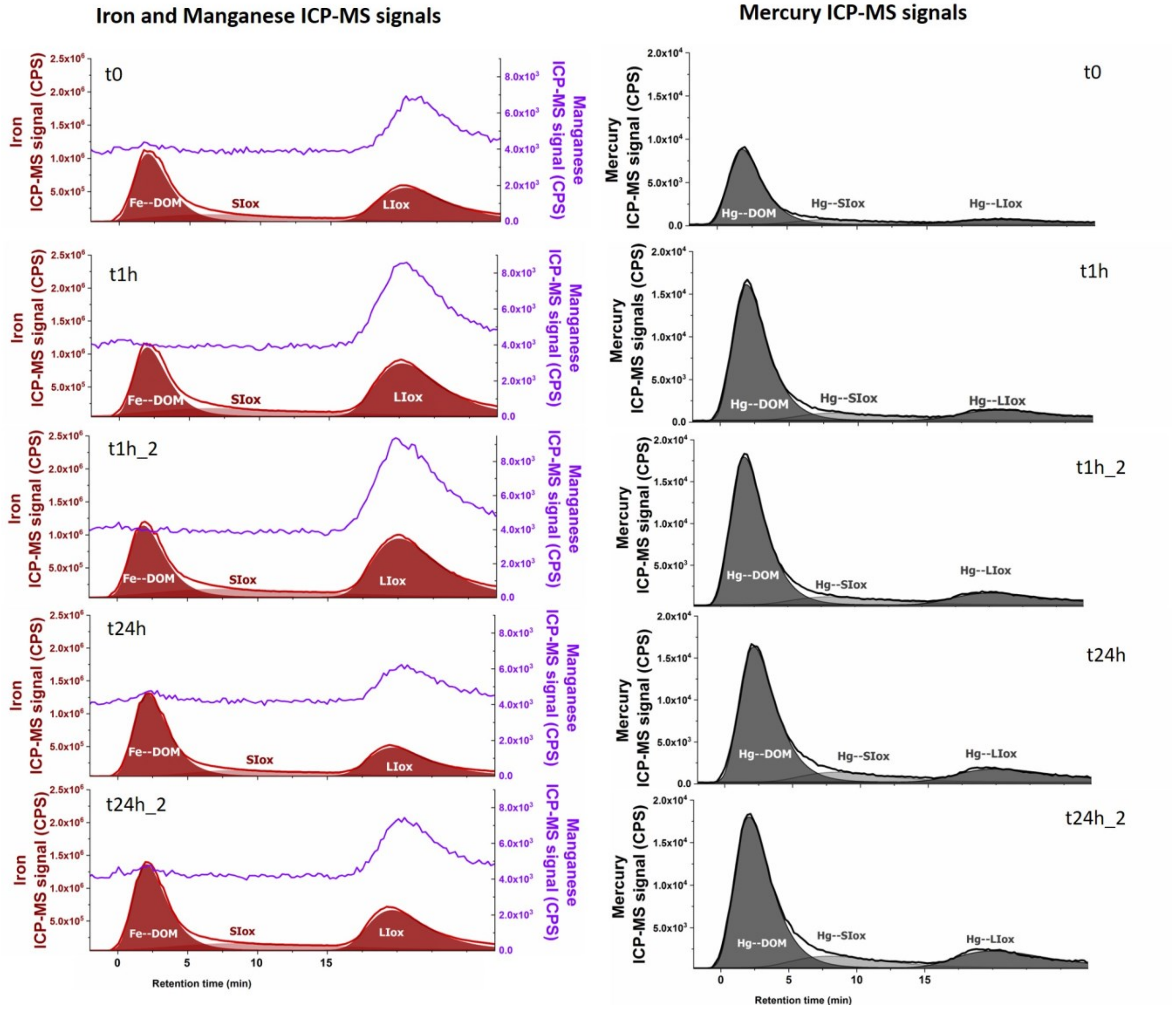

Figure S9. Effect of equilibration time after spiking of 100 nM Hg on the size distribution of colloidal metal (^{55}Mn, ^{56}Fe) and bound Hg (^{202}Hg) measured by AF4–ICP-MS of water sampled from Petrozadovosk Bay. Small changes in size distribution and recovery of SIox over the time and variability in LIox and Mn colloids were observed. This relays on the non-optimized conditions for large inorganic colloids following our procedure, focused on < 17 nm components. The quantity of Hg bound to colloidal DOM (Hg–DOM) was relatively constant for equilibration time of 1h and 24h. This indicates that 1) Hg has equilibrated at least with relatively strong binding sites of humic substances after 1h and 2) the recovery of inorganic colloids had little influences on the measured Hg–DOM. Injection performed immediately after spiking (no equilibration, t0) showed lower quantity of Hg–DOM.

Cossart, T., Garcia-Calleja, J., Worms, I.A.M., Tessier, E., Kavanagh, K., Pedrero, Z., et al. (2021). Species-specific isotope tracking of mercury uptake and transformations by pico-nanoplankton in an eutrophic lake. *Environmental Pollution* 288. doi: ARTN 11777110.1016/j.envpol.2021.117771.

Worms, I.A.M., Chmiel, H.E., Traber, J., Tofield-Pasche, N., and Slaveykova, V.I. (2019). Dissolved Organic matter and associated trace metal dynamics from river to lake, under ice-covered and ice-free conditions. *Environmental Science & Technology* 53(24), 14134-14143. doi: 10.1021/acs.est.9b02184.
